# Vocal learning: Beyond the continuum

**DOI:** 10.1371/journal.pbio.3000672

**Published:** 2020-03-30

**Authors:** Pedro Tiago Martins, Cedric Boeckx

**Affiliations:** 1 Section of General Linguistics, Universitat de Barcelona, Barcelona, Spain; 2 University of Barcelona Institute of Complex Systems (UBICS), Barcelona, Spain; 3 Catalan Institute for Research and Advanced Studies (ICREA), Barcelona, Spain

## Abstract

Vocal learning is the ability to modify vocal output on the basis of experience. Traditionally, species have been classified as either displaying or lacking this ability. A recent proposal, the vocal learning continuum, recognizes the need to have a more nuanced view of this phenotype and abandon the yes–no dichotomy. However, it also limits vocal learning to production of novel calls through imitation, moreover subserved by a forebrain-to-phonatory-muscles circuit. We discuss its limitations regarding the characterization of vocal learning across species and argue for a more permissive view.

## Abstract 2

Vocal learning is the capacity to modify vocal output on the basis of experience, crucial for human speech and several animal communication systems. This Essay maintains that the existing evidence supports a more nuanced view of this phenotype, broadening the set of species, behaviors, and factors that can help us understand it.

## Introduction

Humans (and no other species) have language. An important component for language is speech, which refers to the auditory/vocal medium we use to communicate linguistic units among ourselves, and apart from auditory perception and processing, it requires a vocal tract with a wide range of possibilities, such as ours (but not necessarily exactly like ours [1]), and the capacity for vocal learning. Vocal learning broadly construed is the ability to modify vocal output on the basis of experience. Unlike language, however, vocal learning is not unique to humans: it is present in several, distantly related species. There are different ways in which species modify their vocalizations (see Box 1). This could involve either a modification of an aspect of vocalization (as long as it can be shown that such a modification is learned from experience, usually auditory but not necessarily so [2]) or the production of novel vocalizations altogether.

**Box 1. Simple vocal learning typology**

When looking at vocal abilities, there are ways of teasing apart which behaviors require some form of learning and which don't. There is a major split between vocalizations that are innate, whose employment does not depend on experience, and those that do require experience and go beyond the innate repertoire. The former kind of vocal behavior is common to most animals. It includes crying and laughing [3], for example, and does not require experience (though see [4] for how experience influences prosodic aspects of crying). The latter kind is less widespread, and it consists broadly of three subtypes, following [5]:

- ability to associate a sound with a behavioral response (example: dog [*Canis familiaris*] response to human commands)
- ability to learn the context in which a vocalization can be used (example: vervet monkey [*Chlorocebus pygerythrus*] vocalizations in response to predators)
- ability to modify vocalizations on the basis of experience (example: birdsong), which can converge or diverge from a model

The behavior that interests us here is vocal production learning, which is what most researchers refer to when they refer to vocal learning. But what constitutes vocal production learning as a phenotype is far from agreed upon by researchers, both in contrast to the other subtypes and on its own. This naturally affects which species are considered capable of it.

Vocal learning is indeed a very productive area of study across disciplines [6]. Species that display vocal learning abilities are a relevant source of information on the nature and evolution of language in humans, chiefly regarding phonological aspects [7]. However, not everyone agrees on what constitutes vocal learning as a phenotype, and this greatly affects how work on vocal learning is carried out.

### The “canonical” list of vocal learners

There is a general trend in the literature (e.g., [8]) that limits vocal learning only to species that can produce novel calls through imitation, subserved by a direct connection between the forebrain and phonatory muscles (e.g., the larynx in mammals or syrinx in birds). This has led to a canonical list of vocal learning species. It comprises three bird orders and some mammals. The birds—by far the longest list when counting individual species (in the thousands [9])—comprise songbirds (Passeriformes), parrots (Psittaciformes), and hummingbirds (Trochiliformes) [10, 11]. The mammals include humans, some cetaceans [12, 13], pinnipeds [14, 15], elephants [16], and bats [17, 18]. Birds are considered closest to humans in vocal learning abilities, even though they are phylogenetically the most remote. Humans are the only primate uncontroversially considered to be vocal learners, whereas nonhuman primates are considered of little relevance in this regard. Refinements to this list usually consist of looking inward for finer distinctions in the families already established (e.g., bats [18] or parrots [19]), and rarely outward.

It is possible that this focus on imitation and novel vocalizations is due to it being the clearest case of something being “transmitted” and then “learned.” That is, it could be that for those who put a premium on imitation (e.g., [8]), learned (as opposed to “innate”) entails that there has to be imitation of something that wasn't there before in any form (in the repertoire), as opposed to improvisation or other ways in which sounds in a repertoire can change (for example, through social feedback or modifications of aspects of calls that do not entail an entirely novel output).

It is also possible that the appeal of “neuro-reductionism” (to virtually equate a behavioral phenotype with a neural implementation [20]), might have had an influence in the establishment of this take on vocal learning. The existence of a direct connection from forebrain to phonatory muscles, allowing for fine control of those structures, is indeed an appealing idea on which to build.

There is, however, empirical evidence of vocal learning abilities in other species outside of the canonical list. Such evidence is usually behavioral and not an attempt to show direct forebrain control of phonatory muscles. Indeed, there is work questioning that such a connection is a necessary condition for vocal learning in the first place [21, 22].

### The vocal learning continuum and beyond

In light of this, we think it worth discussing the “vocal learning continuum hypothesis” (VLC) [11], which categorizes species along a continuum of increasing vocal learning complexity. This is a valuable idea that goes against the traditional dichotomous view of vocal learning, according to which a species is either definitely a vocal learner or not at all. However, it too relies on production of novel calls through imitation, subserved by forebrain control of phonatory muscles, to determine the distribution of vocal learning abilities across species. Such an approach is therefore not representative of the diversity of vocal learning behavior across the animal kingdom [23, 24]. This diversity pertains not only to species for which there is recent evidence of vocal learning but also to the “well-established” vocal learning species, namely birds [25].

Indeed, we find that the way species learn to produce their communication signals should form the set of criteria that makes a species a vocal learner. The exact nature of the vocalizations and the neurobiology are of course extremely important, and they will allow for much more precise evolutionary work, but one must not lose sight of the fact that vocal learning is a behavioral phenotype, with learning as the most striking aspect.

Given this state of affairs, two ways offer themselves for future studies on vocal learning: (1) perpetuating the bifurcation between canonical and “negligible” vocal learning species or (2) turning attention to the behaviors observed and assessing them in the context of a broad sense of vocal learning, as opposed to dismissing them on neurobiological grounds alone.

Recently proposed frameworks and reflections also show some concern with this question and call for more wide-ranging perspectives on vocal learning (e.g., [26–28]).

In the remainder of the present paper, we start by going over the VLC and point out some limitations. We then point to evidence from “noncanonical” species that reinforces these limitations and conclude by outlining an extension to the VLC highlighting approaches to vocal learning that can help overcome them.

## Limitations of the vocal learning continuum

The VLC proposes that species can be placed along a continuum, yielding a gradual as opposed to dichotomous classification [11, 29–31]. The categories in the VLC are as follows: vocal nonlearners, limited vocal learners, moderate vocal learners, complex vocal learners, and high vocal learners.

The motivation for the VLC is that some species cannot be clearly categorized as nonvocal learners or vocal learners (in the all-or-nothing sense), with the mouse (*Mus musculus*) being such a case: they seem to have some form of song (ultrasonic vocalizations), but it is not clear whether they are learned or innate. Some aspects of it, however, seem to be contingent on social feedback, which highlights the role of experience. In addition, species with very impressive but not identical vocal learning abilities, such as songbirds versus parrots, can also more safely be placed a notch apart so that they are not equated and the nuances that distinguish them are not lost.

Although the VLC is a very welcome and important proposal for the study of vocal learning, we find that it has some limitations, which must be overcome in order to achieve a full(er) picture of the vocal learning morphospace and an understanding of its evolutionary history.

Some of the limitations of the VLC are of a conceptual nature, and some are empirical. The conceptual limitations are independent of what the VLC is actually about and, instead, have to do with the validity of establishing a two-dimensional model of a complex trait, which had to evolve. The empirical limitations have to do with applying the model to vocal learning specifically and how its predictions don't pan out, for different reasons.

### Bidimensionality

Because vocal learning categories are determined by the existence and strength of a particular brain circuit in the VLC, this makes it a bidimensional system (see [28] for some recent discussion on the same issue).

The particular brain circuit is, to put it in simple terms, a direct connection from the forebrain to phonatory muscles, and it is thought to be present in some form in canonical vocal learning species. The Kuypers/Jürgens (KJ) hypothesis [8, 32] posits that such a connection is necessary for the kind of motor control that is required for vocal learning, and the VLC tacitly relies on it. This idea had already been made popular before (e.g., [33], among others), but it was perhaps made more widespread in the work of Jarvis (e.g., [11, 34]), and Fitch (e.g., [8, 35]), who named the hypothesis after two scientists who made important contributions to primate neurobiology [36–38]. In other words, even though the VLC is a more nuanced conception of how to ascribe vocal learning across species because it allows intermediate steps, it is still limited in the sense that it has the forebrain-to-phonatory-muscles connection as the sole predictor and allows for variation only in that dimension. It is relevant here to recognize the role of a direct forebrain-to-phonatory-muscles connection as a necessary ingredient in the VLC; proponents of the VLC are of course well aware that it alone cannot explain away vocal learning as a whole. Other abilities and traits are involved, such as auditory learning [11], but the VLC is not concerned with them.

An analogy to the bidimensional nature of the VLC would be a slider in a physical machine or a computer program that controls a parameter, and by sliding it back and forth, the output is changed. In this case, the parameter would be the strength of a forebrain-to-phonatory-muscles connection, and the output would be “less” or “more” vocal learning. If the slider is at position 0, we get no vocal learning. If the slider is at the maximum value, we get “high-end” vocal learning.

There are two ways in which this bidimensionality is problematic. The first is that it leaves out capacities and constraints at other levels of analysis [18, 24, 28], which might or might not go hand in hand with this brain circuit. This is well captured by the following questions, taken from [28]: (1) What makes a species a vocal learner? (2) When is vocal learning employed? (3) How can vocal learning be expressed by the organism? (4) Who (else) is capable of vocal learning? And (5) why did vocal learning evolve?

Recent empirical evidence shows that species that do not or are thought to not have relevant forebrain control of phonatory muscles can be vocal learners. This brings to the fore other ways in which species may achieve what is in effect vocal learning behavior. Testing of species whose vocal learning capacities are unknown or supposed not to exist still yields surprises.

The other problem is that if a goal (or even the main goal) of comparative work is to derive information about the evolution of traits and species, we cannot expect a single aspect (e.g., a single genetic change or a single brain connection) to offer a realistic picture of how the trait evolved [39]. Even if the empirical evidence established that only vocal learning species in any one sense consistently have a certain brain connection and vice versa, evolutionary aspirations would still require a more complex explanation. In the realm of complex traits, there is always a cascade of effects with far-reaching implications [40]. It is also the case that even homologous behaviors don’t necessarily share a neural mechanism: there can be genetic changes affecting circuitry with no change in behavior [41].

### More on brain wiring

The discovery of particular wiring (see Box 2) made it possible to attempt a principled, brain-based separation of strictly innate calls in a way that’s shared among all mammals tested from calls that are controlled volitionally. However, even in the very strict sense of learning of novel vocalizations through imitation, it is not known beyond doubt that this is a necessary condition. For example, there are reports of learned, voiced calls in the orangutan (*Pongo* spp., a species that supposedly lacks the relevant connection [42]). It is also not clear whether the connection is sufficient (within reason) either; mice (*M*. *musculus*) apparently have the circuit but do not produce novel sounds through imitation [29, 30], and perhaps more interestingly, recent work shows that female zebra finches (*Taeniopygia guttata*), which do not produce learned song, have “male-like” song pathways [43], so the narrative is not totally compelling. Furthermore, there is work showing the involvement of other structures and pathways in the learning of vocal behavior in a relevant manner, such as the cerebellum [44], the periaqueductal gray (PAG) [2], or the ventral tegmental area (VTA) [45]. It is also not entirely clear why vocal learning, a phenotype whose most interesting aspect is arguably the learning part, must be limited to a certain kind of vocalization, namely the kind that requires fine control of the phonatory muscles (what is usually referred to as “phonation”). Moreover, it is important to bear in mind that any one connection does not exist in isolation; each brain region involved will be part of several other connections, each with its own complex evolutionary history.

**Box 2. Two major pathways**

There are two major pathways believed to be specifically involved in vocal behavior: a general, “primal” one that is associated with all vocalizing animals and, in addition, a more specific one that is associated with vocal learners.

The primal pathway goes from the anterior cingulate cortex to the PAG, to the reticular formation of the pons and medulla, and from there to the phonatory neurons [3]. It seems that the PAG pathway is not involved in vocal motor coordination but, instead, is responsible for initiation and intensity of what is in effect a vocal reaction. It is not involved in its patterning.

Besides this pathway, used for “reactive” or “affective” vocalizations, it is hypothesized that vocal learners also have a direct connection from the laryngeal motor cortex to the nucleus ambiguus (Am) and, from there, to the phonatory muscles. In birds, similar pathways are thought to exist. There is a connection from the dorsal medial nucleus of the midbrain (DM) to the 12th nerve nucleus, which controls the syrinx. This is the vocalization pathway analogous to the PAG pathway in, say, humans. In vocal learning birds, there is also a connection from the robust nucleus of the arcopallium to the 12th nerve nucleus [46]. Nonvocal learning birds are thought to not have such connections (e.g., pigeons [*Columbia livia*] [47], but evidence is scarce). This direct telencephalic connection in birds is analogous to the cortical connection in humans.

Not much is known about the presence of these connections in some of the families included in the canonical list of vocal learners (e.g., cetaceans and pinnipeds).

This association between medial pathways and innate vocalizations, on the one hand, and cortical pathways and vocal learning, on the other, has become established in the literature, but the claim made by the KJ hypothesis is not without challenges [21, 22]. Most relevant here are perhaps the criticisms by Lameira [22] because they are presented in light of comparative evidence. One argument has to do with attribution: the work by Kuypers and Jürgens does not show or entail what the hypothesis states. For example, Kuypers [36] is assumed to have shown that great apes did not have the required forebrain-to-larynx connection, when in reality, he did in fact identify it in a chimpanzee (*Pan troglodytes*) subject, and Jürgens [38] used monkeys and not great apes in his work. This casts some doubt on our understanding of direct vocal control in chimpanzees and, potentially, other primates. The second argument has to do with evidence against what the hypothesis predicts: nonhuman primates should not in any way display vocal learning. Yet evidence for primate vocal learning is accumulating (see, e.g., [22, 24, 48]). We go into more detail in section S1_Text. Evidence in the opposite direction also exists: mice seem to have the required machinery, yet they are not vocal learners in the KJ sense [29]. From a neurobiological point of view, this should mean that either this particular connection is not necessary in principle for vocal learning or that nonhuman primates actually have it and that interpretations of the few data on this matter are incorrect. The third argument has to do with the very mechanical requirements the KJ hypothesis put forward for vocal learning, which rely heavily on vocal fold control. Also in the formalization of the VLC, this is assumed explicitly: “Vocal learning is the ability to modify the spectral and syntactic composition of vocalizations generated by the vocal organ (larynx in mammals or syrinx in bird)” [29]. This requirement leaves out supralaryngeal vocal production—equivalent to voiceless consonants in humans. These vocalizations, which in humans are the most widespread [49], involve the control of several structures above the “vocal organ,” such as the lips and jaw, and are used as well to expand the vocal repertoire. This might seem like a minor point, but it is worth emphasizing that human language, through speech, makes use of both voiced and voiceless sounds in all known languages. It is also the case that whispered speech, for the most part supralaryngeal, is intelligible, and there is evidence for the use of different acoustic cues in the absence of fundamental frequency [50]. Direct control of phonatory muscles—which produce voiced sounds—alone will leave a great deal unexplained. It has been suggested that, because control of these supralaryngeal structures is clearly present in nonhuman primates, laryngeal control is the extra neurobiological ingredient (a “derived trait” or autapomorphy) that made humans vocal learners [32, 51], but as far as we can assess, this only says something about the sound source and ultimately the acoustics, not about learning and, therefore, not about how ancient or widespread the ability would be in nonhuman species.

It thus seems that, although phonatory muscle control is obviously a very useful ability, relying on the KJ hypothesis alone might not give us a good indication of the basis of vocal learning and how widespread the phenotype is.

### Complexity considerations

The VLC also aims to represent various degrees of vocal learning complexity. But as we will see on at least three counts, it does not do so satisfactorily.

Bengalese finches (*Lonchura striata domestica*) are the domesticated strain of the white-rumped munia (*L*. *striata*). Domestication has been claimed to increase vocal learning complexity: if a “wild” species is already a good vocal learner, it becomes a more complex vocal learner after undergoing domestication [52]. In the case of the Bengalese finch, for example, this happens despite the fact that this bird species was not bred for its song. It is possible that imitation—crucial per the KJ hypothesis and, concomitantly, the VLC—could actually be detrimental to syntactic complexity. Compared with their wild counterparts, Bengalese finches display higher unpredictability and syntactic complexity in their song because not only do they imitate their tutors (partially) but they also improvise, resulting in what is, in effect, lower imitation fidelity [53].

The full “classic” circuit of vocal learning involves a posterior pathway for vocalization and an anterior pathway for learning [34]. Besides those pathways, parrots were discovered to have a “shell” song system in addition to the “core” song system in all vocal learning birds [19]. A larger shell system relative to the core system is associated with parrot species that have more “complex” vocal learning abilities, and vice versa. Moreover, this shell system has mostly intercortical connections, as opposed to the direct connection to the motor neurons characteristic of the core system. It seems, then, that parrot species with a larger shell system have an edge in the VLC, but this edge is not related to the direct connection the VLC rests on. This poses a challenge to the VLC as it stands because it requires the addition of an extra factor (say, adjacent “song/speech” nuclei with intercortical connections or even just “strong intercortical connections”), opening way to a much less restrictive VLC because more factors would be added as needed for capturing differences between species, departing from the bidimensionality we have already alluded to. Indeed, Chakraborty and Jarvis [54] acknowledge it might not be straightforward to reconcile the core/shell system with the VLC.

Finally, in the VLC, humans alone are considered high-end vocal learners, whereas parrots are classified at a level just below, referred to as complex vocal learners. This is purportedly because of the higher syntactic complexity in human vocalizations, but this does not rest on the criteria for categorizing species along the VLC (presence and strength of direct connection to the phonatory organ and imitation). Language complexity need not even be instantiated in vocal behavior; it is well established that the linguistic capacity is the same in sign language (see [55]). It could be that, indeed, humans are the most advanced vocal learner, but this is not possible to discern from the criteria used in the VLC. It might have more to do with the process of cultural transmission and not with anything “vocal” [56]. In a manner similar to Bengalese finches, it has been hypothesized that the increased prosociality that characterizes domestication allows for the jump in complexity to take place (see [56] for discussion).

## A more permissive view

Recent work has a more wide-ranging view of what constitutes a vocal learning species and of what plays a role in it. This, we contend, is necessary in order to extend the idea of the VLC and overcome its limitations.

### Imitation and de novo vocalizations are not the whole story

An important step, in our view, is to adopt a view of vocal learning behavior not necessarily focused on imitation.

The production of de novo vocalizations (new in a species repertoire) and, furthermore, doing so through imitation is often taken as the golden standard when assessing vocal learning abilities. This is problematic because imitation is one possible means of displaying vocal learning behavior. Indeed, diverging from imitation is also a common phenomenon in vocal and cultural development [23].

Perhaps a more productive conception of vocal learning is looking at learned vocal behavior as having to be acquired in some manner over developmental time, especially in contingent ways (that is, dependent on experience and not a “certainty” given the initial state of the organism).

There are interesting cases that illustrate vocal development by diverging from the tutor song; that is, by the countering of or lack of imitation. Infant marmosets, for example, develop vocal learning abilities through social reinforcement from parents, not imitation. This leads to more control of the vocal apparatus, which allows them to produce lower entropy calls [24, 57].

Canaries (*Serinus canaria domestica*) trained on atypical song imitate it at first but, when reaching maturity, shape it into the species-specific song they were never exposed to [58]. Another well-known example is the de novo emergence of zebra finch song not by imitation but, instead, by the approximation of wild-type song over a couple of generations by birds reared in isolation, with no exposure to singing tutors [59].

Evidence of this kind is good indication that vocal learning is not driven (solely) by imitation and that vocal learning ability is characterized also by behaviors that suppress imitation.

### Evidence from species outside of the canonical vocal learners list

Opening up to more-permissive definitions of vocal learning goes hand in hand with opening up to the study of more species. A decent amount of evidence for vocal learning outside of the canonical list has been put forward, especially in recent years, with primates as the most representative of this trend, and some work on rodents. They moreover deserve special attention because there is resistance to taking this kind of evidence into account. Other species are more quickly accepted, perhaps because they employ imitation, and neurobiological information on these species is given a great deal of importance, given its scarcity. A good example of this is the African elephant (*Loxodonta africana*), which quickly entered the accepted list of vocal learning species [16].

The logistic difficulties in keeping and studying larger species as opposed to birds and other (usually smaller) species might also bias positions against, say, primate vocal abilities, leading to a situation in which absence of evidence might be mistaken for evidence of absence. Rodents present challenges on their own (e.g., several species produce ultrasonic vocalizations, which pose further challenges, and there is a lot of interindividual variation [60]). Studies on birds have unsurprisingly dominated vocal learning research (see data in [28]). Great ape language acquisition projects (e.g., [61]) might also have contributed to this state of affairs, given their varying goals and approaches, which usually had to do with finding some form of human language, as well as difficulty in interpreting their results. In Box 3, we summarize some evidence that we think deserves, at the very least, attention if vocal learning is to be understood as a phenotype that’s characterized by the learned modification of calls, with the exact nature of the calls being an important but not (dis)qualifying feature. A more complete (yet not exhaustive) list can be checked in S1_Text.

**Box 3. Evidence of vocal learning abilities in species outside of the canonical vocal learners list**

Primates and rodents are not usually considered to be vocal learners, yet they display behavioral traits that fall within vocal learning in some sense. This is at odds with what circuitry is thought to be required for vocal learning behavior under the KJ hypothesis and the vocal learning continuum and warrants a rethinking of what is really known about the neurobiology of vocal learning. Moreover, some of these species are as well understood as others that do count as vocal learners in literature, warranting in this case a rethinking of the motivations for including some species but not others in the canonical list. Turning first to primates, both monkeys and apes display relevant behavior. Marmosets, a New World monkey who engages in turn taking [62], uses different proportions of affiliative call types depending on social distance [63], as well as loudness relative to physical distance [64]. They can also convey identity through aspects of their calls [65]. Their calls change from infancy into adulthood, much like in humans, and limiting parent feedback disrupts this development [66]. Several Old World monkeys display relevant vocal learning abilities. Diana monkeys show call converge in social interactions [67]. Campbell's monkeys, also a turn-taking species [68], sequence the sounds in their repertoire in a nonrandom way in different situations [69]. Rhesus monkeys have a juvenile period of volitional vocalizing, disappearing once adulthood is reached [70]. Apes show striking vocal learning abilities. Orangutans can learn voiced calls [42, 71] and whistles [72, 73]. They also employ “instrumental gesture calls,” whereby they volitionally use their hands or leaves in front of their mouth to lower the maximum frequency of calls [74, 75]. Gorillas [76] and chimpanzees [77] have also been shown to display vocal learning behavior. Turning now to rodents, there is promising evidence for vocal learning as well. Mice, who produce complex ultrasonic vocalizations, display variation in syllable type, which can distinguish between individuals [78]. They have also been shown to require feedback to maintain certain features of their song [30] and of changes in song development [79]. There is also Alston’s mice, who engage in vocal bouts, which because of their length and patterning, have been deemed worthy of being called song [80, 81]. See S1 Text for an expanded list of species and abilities.

We believe that evidence of the kind we review here has only been neglected because of its nonconformity with the KJ hypothesis. We find that one way of getting a full picture of vocal learning is placing the focus on observing the behavior, without preconceptions of what should allow it, and then proceeding with the mechanisms. As put by Krakauer and colleagues [20]: “The neural basis of behavior cannot be properly characterized without first allowing for independent detailed study of the behavior itself.”

The relationship between a neural structure and a behavior is not one of explanation of the behavior [20]. This is the case even if that relationship is consistent, which in the case of vocal learning and according to evidence we reviewed, it might not be. Although some neuronal implementation will of course be in place, there is no indication that the vocal learning phenotype can be equated with a particular one given that there is not even a consensus on what the behavior encompasses. In the face of paradoxical evidence (e.g., primates displaying vocal behavior they are not “supposed to” have), rejecting the behavioral evidence instead of revising the neural hypothesis will not lead to understanding of the trait. It is in this sense that we think it is important to have behavior as the entry point to the study of vocal learning. If the VLC is extended beyond the specific neural substrate that is taken to allow the direct control of the vocal organ, we could gain a better understanding of the phenotype.

## Tree of vocal learners with a focus on behavior

**Fig 1:**
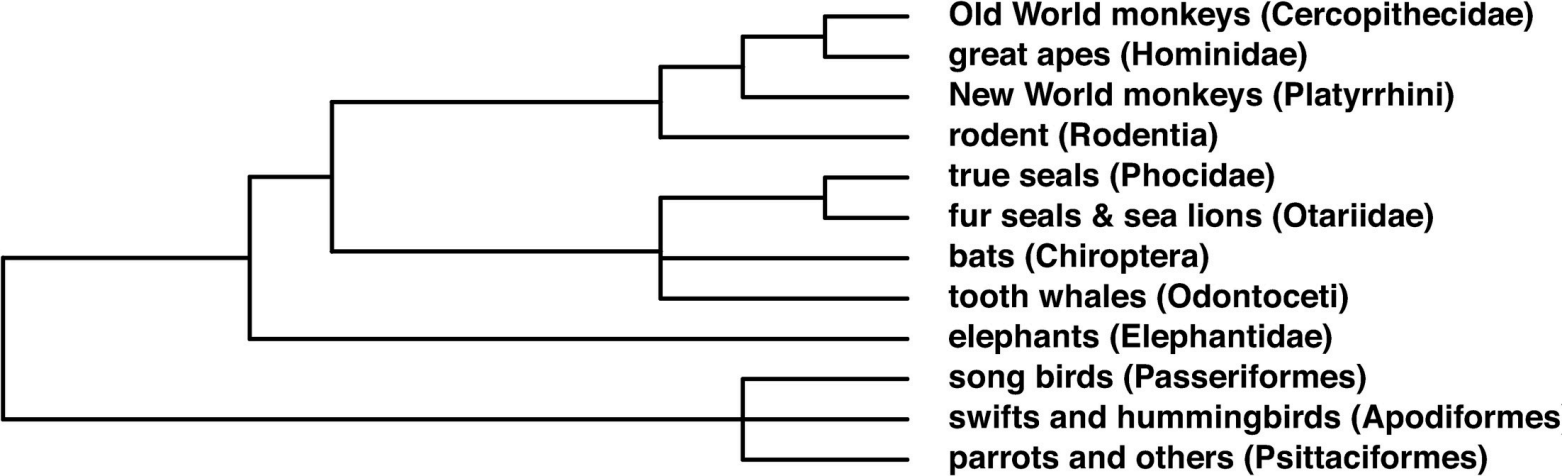
Tree of vocal learners with a focus on behavior. Differences between this tree and the canonical tree are reflected in the presence of primates and rodents. The inclusion of whole families or orders is made under the assumption that all members thereof are at least worth studying and is not a claim about their actual vocal learning abilities, which are an empirical question. Common and scientific names from NCBI (https://www.ncbi.nlm.nih.gov/taxonomy). Tree built with taxize R package [82]. NCBI, National Center for Biotechnology Information.

Taking all the evidence available into account, and placing the focus in the behavior observed, we believe a more accurate “vocal learners list” (albeit with some tentative cases) would be the one we sketch in Fig 1.

We can see in this new list that it is possible to reduce the gap between us and the other vocal learners in a principled way. Although a direct forebrain–larynx connection is maybe not shared, there is much that is shared: similar patterns of early postnatal vocal development [66], volition [24], both voiceless and even voiced calls [42], socially reinforced vocal production, etc.

**Fig 2:**
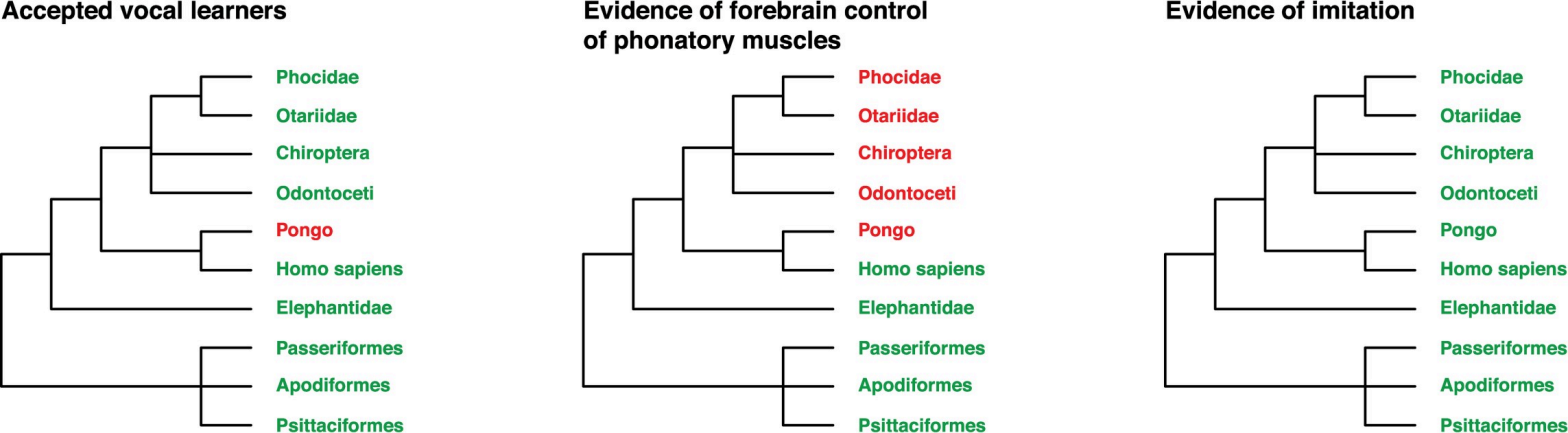
Comparison of simplified phylogenies of vocal learning: The canonical list with the addition of the orangutan (*Pongo*) for contrast. Key: green indicates species or orders considered to conform to the criterion on each tree, and red indicates species or orders that are considered not to. Left: list of accepted vocal learners. Center: list of species for which there is evidence of a direct connection between forebrain and phonatory muscles. Right: list of species for which there is (some) evidence of vocal imitation.

The canonical list of vocal learners, although much more manageable, is in effect a list of species for which there is, on the one hand, evidence of imitation and, on the other hand, evidence of direct connection from forebrain to phonatory muscles or an assumption of its existence (Fig 2, left). Assumption of its existence relies on two other assumptions: that this circuit is crucial for vocal learning and that nonhuman primates cannot have this circuit. But the fact of the matter is that there is no demonstration of this circuit for some species routinely considered vocal learners (Fig 2, center) that do show vocal imitation (Fig 2, right). In terms of evidence, there is nothing separating, say, orangutans from seals: there is evidence of imitation for both species, yet only one is an accepted vocal learner. Although one could argue about the strength of the evidence for some species over others, as well as ease of elicitation or perceived quality of the behavior, this disparity in the way different species are categorized seems to be stipulated by the KJ hypothesis and therefore warrants further reflection.

In previous work, we suggested the term “sound production” learning as opposed to vocal (production) learning as what might be a better term for defining the capacity we are interested in here, given the association in the literature of vocal with the phonatory muscles [83]. This might allow for a more encompassing definition, regardless of the mechanics involved. This would dilute a distinction that, as a characterization of the behavior, is not very relevant: if a species can change its repertoire, be it through imitation or not, the exact structures of the vocal tract that are used to doing so are not grounds for a big divide in the classification of the behavior, at least not with regard to learning. This is similar to what happens with the exact brain structures used in different vocal learning species: the fact that birds have no cortex and no larynx but, instead, telencephalon and syrinx does not warrant a strong divide as far as behavior is concerned, and indeed, birds are considered the prime model species for studying vocal learning in humans. Using the term sound production learning could lead to the inclusion of sound sources not limited to the vocal tract or other orofacial structures. In our view, it depends on the degree to which vocal learning is grounded in behavior and learning as opposed to the sound sources and the pathways expected to control them.

## Vocal learning contiguum: An outline

Focusing on just one measure of any one cognitive trait (that is, a “two-dimensional” continuum [28]), seems to be an attempt to find neat cognitive phylogenies (in the sense of Fitch and colleagues [35]) for what is a complex behavior (see [84] for discussion). This becomes an easier task if vocal learning is reduced to a single circuit because it allows one to conceive of single events (nodes in a phylogeny) that confer the behavior to a species and its descendants (e.g., a whole order of birds). If other factors are considered, however, different cognitive phylogenies could be devised. If we ask all the questions posed by Lattenkamp and Vernes [28] for each species, we will see that there will be gaps, but we will see as well that each species provides answers to at least some of the questions. This is therefore in our view an adequate list given all the evidence.

**Fig 3:**
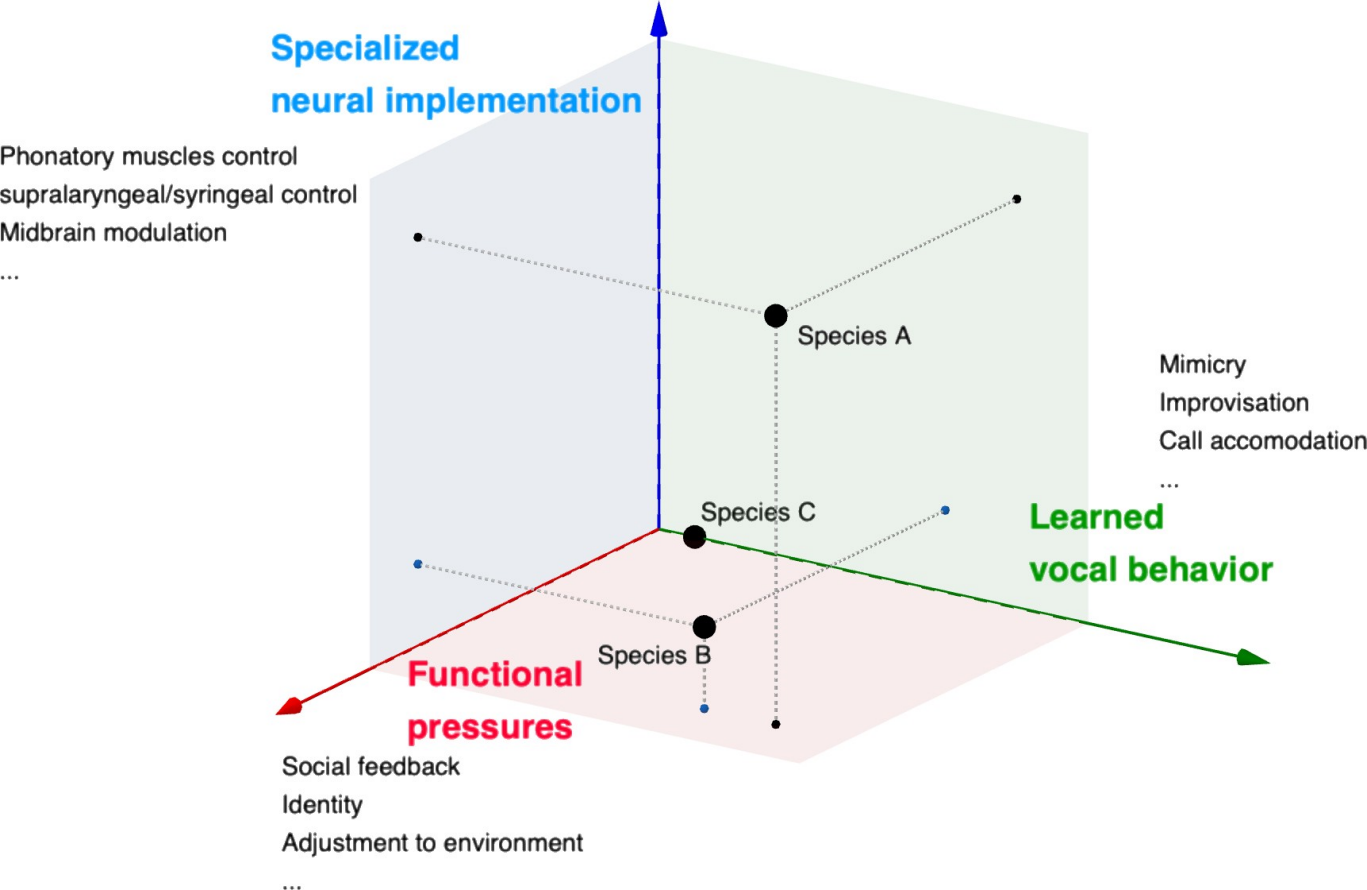
Outline of a vocal learning contiguum. Three main dimensions are considered: learned vocal behavior (green), functional pressures (red), and specialized neural implementation (blue). Examples are given to different factors that can be included in each of these dimensions. Black dots represent hypothetical species placed in the morphospace according to evidence. Species A displays vocal learning abilities and is thought to have a particular neuronal implementation in place, as well as subject to considerable functional pressures that help shape the behavior. Species B is a vocal learner, but evidence for a particular specialized implementation is scarce. For species C, there is no behavioral evidence of vocal learning, and no specialized neural implementation for vocal learning is thought to be present (and concomitantly no functional pressures).

In Fig 3, we outline an extension of the VLC, which we call the vocal learning contiguum, to capture the notion of a space of neighboring and overlapping factors, as opposed to a linear scale (as in the VLC). In this conception, vocal learning is understood as a morphospace, and a species can be represented as displaying vocal abilities of a certain type without a necessary association with either a specific neural implementation or a specific set of functional pressures. If groups of species congregate in particular areas of the morphospace, one could take this as a good indication of which factors help shape them as vocal learners and to which degree. This is in a way a simplification of the several factors that contribute to a complex behavior. A more realistic picture would comprise several dimensions. Here, for representational purposes, we collapse them into three: evidence of vocal learning behavior in a broad sense, evidence of a specialized neuronal implementation, and evidence of functional pressures (environment, social feedback [auditory or not], etc.) that help shape the behavior. The “position” of each of these species along each axis can be understood as identifying how much evidence there is for this factor playing a role. Each of the axes, which can be understood as “macrodimensions,” can be further decomposed, and each of the subcomponents would also be subject to different factors.

The choice of “contiguum” for our conception of vocal learning is intentionally similar to “continuum,” used in the VLC. We do not intend ours to be understood as something completely separate from the VLC but rather as an extension. In this sense, instead of a line, or even a ladder, taking us from category A to B to C and so on, we imagine a morphospace in which different forces bring a species closer to the behavior or pull apart. Like the VLC, we acknowledge the nondichotomous nature of vocal learning abilities. But we go beyond it in considering more factors than VLC for determining such abilities. The VLC would in effect be a vector in our contiguum: one of several “forces” pushing a species toward one or another phenotype. We name it “specialized neural implementation” in Fig 3. The female zebra finch, for example, which possesses pathways that some considered conducive to vocal learning [34], lacks the behavior readily displayed by males [43], which goes to show that a specific neurobiological pathway cannot be used as a predictor.

Other authors have recently put forward ideas and frameworks that we believe go in a similar direction to ours. Lattenkamp and Vernes [28] and Vernes and Wilkinson[18], though focusing on bats, call attention to the importance of behavioral, developmental, social and motivational, neurobiological, and ecological factors that play a role in vocal learning. We think this is the right approach to take and that it can be extended to other species: other species can qualify as vocal learners if we accept that neuronal wiring is just one aspect contributing to vocal learning.

These questions of the kind posed by these authors [18, 28], in our view, follow a pedigree of influential work that has ultimately shaped biological research and contributed to a better understanding of cognition. We are referring here specifically to Tinbergen’s four questions [85], which ask about mechanism, evolution, ontogeny, and function, and Marr’s three levels of analysis [86]: the computational, algorithmic, and implementational levels. These frameworks have forced researchers not be to tied to any one level of description, and keeping all of them in mind when seeking understanding contributes to what Krakauer and colleagues [20] have recently called a pluralistic notion of neuroscience.

Wirthlin and colleagues [26] have a very recent proposal whereby vocal learning can be understood as being made up of different subcomponents, or “modules,” and they start by looking at three: vocal coordination (ability to flexibly modify the temporal production of vocal output), vocal production variability (ability to dynamically change acoustic variability throughout development), and vocal versatility (repertoire size versus degree to which it can be modified with experience). Though not exhaustive, these three modules encapsulate several aspects commonly associated with the vocal learning phenotype. Species can be placed along “axes” for each module, and precise comparative and evolutionary characterizations can be attained.

These proposals differ in their details but find commonality in advocating for a multidimensional view of the vocal learning phenotype, which will lead to a more complex but also more accurate representation of its distribution and characteristics. Marrying ideas of this kind with evidence of the kind we review, we believe a more permissive view of vocal learning will start taking shape, encouraging further comparative studies.

## Conclusions

Like other aspects of cognition, vocal learning is a mosaic, made up of different parts. The shared aspects of it should make this even less controversial than, say, language because no one can claim—as they do for language—that what other species have is very different and hard to compare to what we have.

As with any trait, an encompassing view of vocal learning makes it harder to pin down its evolutionary history and the mechanisms behind it. But reducing it to a very specific phenotype and mechanism limits the scope of comparative work, and although it might give the impression that the phenotype becomes more tractable and well-defined, it invariably forces one to subscribe to a very narrow conception that relies on a single driver. Language in general is a good (if extreme) illustration of this. Attempts have been made to reduce language to a very narrow phenotype in order to better study it (e.g., [87]). However, such approaches prevent comparative work almost by definition. As far as we can tell, reductionist views of the language phenotype have not been fruitful and have led to implausible scenarios for the evolution of language [39, 88]. The case of vocal learning is not as extreme because virtually any definition of it yields more than one species with the trait, therefore allowing for some comparative work (although according to the VLC, humans are the only high-end vocal learners). Including more species under the umbrella of any one phenotype should not be a goal in and of itself, but there is much room in our view for casting a wider net and capturing the behaviors that are now being uncovered in other species.

## Supporting information

S1 Text Evidence of vocal learning in species not traditionally considered to display vocal learning abilities. (PDF)

## Abbreviations

Am: nucleus ambiguous
DM: dorsal medial nucleus of the midbrain
KJ: Kuypers/Jürgens
NCBI: National Center for Biotechnology Information
PAG: periaqueductal gray
VLC: vocal learning continuum hypothesis
VTA: ventral tegmental area
