# Supplementary material for: Vocal learning: Beyond the continuum
Source: PLoS Biol. 2020 Mar 30;18(3):e3000672. doi: 10.1371/journal.pbio.3000672 (PMC7145199; doi:10.1371/journal.pbio.3000672)
Supplement: S1 Text — (PDF) [file pbio.3000672.s001.pdf]

Supplementary information for

Martins PT, Boeckx C. Vocal learning: beyond the continuum.

PLoS Biology. 2020. doi:10.1371/journal.pbio.3000672

S1 Text

Pedro Tiago Martins[1,2,*] and Cedric Boeckx[1,2,3]

[1]Section of General Linguistics, Universitat de Barcelona

[2]University of Barcelona Institute for Complex Systems (UBICS)

[3]Catalan Institute for Research and Advanced Studies (ICREA)

Description

This supplementary file lists evidence of vocal learning in species not traditionally considered to display vocal learning abilities, namely primates and rodents. It offers a more complete (yet not exhaustive) list of species we find relevant in the study of vocal learning. This list is summarized in Box 3 in the main paper [1].

Abbreviations: BA, Brodmann's area; VLC, Vocal learning continuum hypothesis.

1 New world monkeys

Marmosets use different proportions of affiliative call types depending on social distance, in a way that arousal levels alone cannot predict [2]. They also control loudness relative to distance, which they assess from the loudness of their vocalizing conspecifics [3]. There are also aspects of marmoset calls that convey gender and identity [4]. Marmoset calls change during the transition from infancy into adulthood. Even though part of this change is explained by growth, but also parental feedback, much like in humans [5]. They do this not through imitation but rather through the experience-dependent increase in the control of the vocal apparatus that allows them to more consistently produce low entropy (adult-like) calls [6]. Indeed, limiting parent feedback disrupts this development [7].

Other features of the vocalizations are unaffected by experience, but instead by growth [8]. It has recently been shown that adult marmosets also spectrally modify their calls to avoid clashing with interfering sounds [9], and another study shows call structure variation between different populations [10]. Moreover, recent work shows that marmoset calls, not unlike human speech, are made up of several small units, instead of longer discrete ones [11].

Marmosets engage in vocal turn-taking [12, 13], in a way similar to humans [14]. If evidence described in this section is taken into account, there seems to be a connection between turn-taking and vocal learning, reflected mainly in humans and non-great apes (though it is possible that other

primate orders will reveal interesting in this regard as well). Recent work highlights the importance of turn-taking behavior in the emergence of controlled vocalizations. The auditory input that "participating" individuals receive from one another leads to changes in their own vocalizations, and the reciprocation and continuation of the exchange ultimately shapes vocal behavior [15–17].

Another species of marmoset, the pygmy marmosets (*Cebuella pygmea*), has also been studied. Similarly to the common marmoset, they are also turn-takers [18]. They display within-subspecies vocal differences [19]. Individuals with different call structure converge when paired, with long lasting effects [20]. Pygmy marmosets also change call structure under new social environments [21].

Cotton-top tamarins (*Saguinus oedipus*) change several aspects of their vocalizations in when noise is present in their environment. [22].

2 Old world monkeys

Diana monkey (*Cercopithecus diana*) show call convergence in social interactions [23]. Campbell's monkey (*Cercopithecus campbelli*), also a turn-taking species [24] display an impressive repertoire for several different situations, with non-random transition probabilities when sequencing their calls [25].

Rhesus monkey (*Macaca mulatta*) can learn to vocalize in response to (experimenter) visual cues [26]. They also have a juvenile period of volitional vocal control, which seems to disappear when adulthood is reached [27].

Japanese monkeys (*Macaca fuscata*) match acoustic features of their calls to those of the calls they are "replying" to in interactions with conspecifics [28]. Olive baboons (*Papio anubis*) adapt their vocalizations with respect to their environment, with longer, lower frequency calls in closed habitats [29]. Mandrills (*Mandrillus sphinx*) approximate their calls to closer-related conspecifics [30].

3 Apes

Chimpanzees show call convergence: after being moved to a new place, food calls of a chimpanzee population changed towards those of the resident group [31]. This was shown again in wild chimpanzees [32]. This seems to occur even within a group [33]. They also produce grooming-related calls consistent with social learning and not with associative behavior [34]. There are also reports of modification of food call structure according to tree size, with smaller trees (of the same fruit, i.e. this is not a label for a tree type) corresponding to higher pitch size [35]. Chimpanzees are also more likely to produce attention-getting vocalizations if human experimenters and food are present [36]. Interestingly, chimpanzees that produce attention-getting sounds (e.g. raspberries) have greater gray matter density compared to individuals who don't, specifically in ventrolateral prefrontal cortex and the left dorsal premotor cortex (roughly corresponding to BA 44 in humans) [37]. This lends support to the idea that these calls — which are voiceless, and therefore are not subserved by vocal fold control — are due to some form of vocal learning, as opposed to innate.

Orang-utans, even those belong to the same subspecies and therefore presumably having the same genetic makeup, sometimes employ different calls in similar situations, so much so that different populations will have a different number of calls in their repertoire. The repertoire size is correlated with the amount of social interactions within each population [38, 39]. There are also cases of acquisition of new sounds. An orang-utan in captivity has been reported to acquire a

human-like whistle, which it had the capacity to modulate [40]. Whistles are completely absent from known (non-human) repertoires, and later more orang-utans were shown to also have learned whistling from conspecifics [41]. There is also evidence for the learning of a voiced call, likely from humans [42], which is more remarkable in a sense, since this should require forebrain projections which orang-utans supposedly lack. This case is not isolated, as another voiced call was later reported in a different individual [43]. A very recent study shows that orangutans are capable of producing sound using a membranophone, which requires vocal fold vibration. Moreover, they do so by producing species-atypical vocalizations, and continue to alter the vocalizations to enhance the output through the membranophone [44].

Orangutans also employ what came to be known as "instrumental gesture calls": they make use of their hands or objects such as leaves to lower the maximum frequency of calls, resulting in size exaggeration. This is a way of exerting vocal control by circumventing the lack of direct control of the larynx they might have, and seems to be a volitional behavior [45,46]. Gorillas (*Gorilla gorilla*) have been shown to produce "raspberries" in some populations but crucially not in others, with no known ecological factors that cause this. In other words, some form of vocal tradition, akin to a dialect, had to be learned and transmitted [47]. There is also a study reporting the volitional production of several "breathing related-behaviors", involving dynamic coordination of breathing, larynx, tongue and lips [48]. In gibbons (*Hylobates agilis agilis*), mother-daughter vocal interactions help the development of species-specific vocalizations [49].

4 Rodents

Rodents is perhaps too broad a category, but is too understudied an order (in terms of number of species) to make bold claims about which families definitely are or are not vocal learners.

The mouse (*Mus musculus*) shows features that may qualify it as a vocal learner, produces complex (ultrasonic) vocalizations, comprising different syllable types and patterning thereof, and also a striking similarity to birdsong when adjusted to the human freq range [50]. There is also variation in syllable type which distinguishes individuals [50]. Whether their songs are innate or learned is a matter of debate (this was, indeed, what sparked the idea of VLC, since it would be hard to be categorical). Cross fostering experiments between strains that produce different patterns of vocalizations yielded individuals whose vocalizations had aspects of both [51], suggesting that calls are innate. However, sub-strains seem to match each other's pitch under certain conditions [52]. Deafening experiments reached the conclusion that mouse song is innate [53]. However, there is work showing mouse require feedback to maintain certain features of their song [54], and of changes in song across development [55].

Alston's singing mice, (*Scotinomys teguina*), engage in vocal bouts, containing sonic and ultrasonic vocalizations, which due to their length and patterning have been deemed worthy of being called song [56]. Recent results show moreover the involvement of a certain degree of cortical control, important for social, turn taking vocal interactions [57].

The common degu (*Octodon degus*), which has some 15 distinct calls in its repertoire [58], can be trained to vocalize in order to obtain food. Interestingly, they have been shown to, after such training, building nested structures with objects, something they do not do without vocal training [59]. This suggests that external experience, perhaps at a certain developmental stage, even if usually absent in normal circumstances, can promote abilities that would suffice to classify a species as vocal learner, or at the very least not rule out the ability. Also, degus exposed to shocks vocalize differently depending on whether they are being observer by a close individual or

a stranger [60], which highlights aspects of social learning in the patterns of vocalization in this species.

The African mole rat (*Fukomys micklemi*), a social subterranean species, has an expanded vocal repertoire when compared to other, solitary species (for example, the silvery mole-rat (*Heliophobius argenteocinereus*), among others) [61]. While this is not evidence of vocal learning beyond doubt, the fact the major difference between these species is how social they are plausibly suggests there social factors are responsible for the large and diverse repertoire of the African mole rat.

References

[1] Martins PT, Boeckx C. Beyond the Vocal Learning Continuum. PLOS Biology. 2020. doi:10.1371/journal.pbio.3000672.

[2] Liao DA, Zhang YS, Cai LX, Ghazanfar AA. Internal States and Extrinsic Factors Both Determine Monkey Vocal Production. Proceedings of the National Academy of Sciences. 2018;115(15):3978–3983. doi:10.1073/pnas.1722426115.

[3] Choi JY, Takahashi DY, Ghazanfar AA. Cooperative Vocal Control in Marmoset Monkeys via Vocal Feedback. Journal of Neurophysiology. 2015;114(1):274–283. doi:10.1152/jn.00228.2015.

[4] Miller CT, Wren Thomas A. Individual Recognition during Bouts of Antiphonal Calling in Common Marmosets. Journal of Comparative Physiology A. 2012;198(5):337–346. doi:10.1007/s00359-012-0712-7.

[5] Takahashi DY, Fenley AR, Teramoto Y, Narayanan DZ, Borjon JI, Holmes P, et al. The Developmental Dynamics of Marmoset Monkey Vocal Production. Science. 2015;349(6249):734–738. doi:10.1126/science.aab1058.

[6] Takahashi DY, Liao DA, Ghazanfar AA. Vocal Learning via Social Reinforcement by Infant Marmoset Monkeys. Current Biology. 2017;27(12):1844–1852.e6. doi:10.1016/j.cub.2017.05.004.

[7] Gultekin YB, Hage SR. Limiting Parental Feedback Disrupts Vocal Development in Marmoset Monkeys. Nature Communications. 2017;8:14046. doi:10.1038/ncomms14046.

[8] Zhang YS, Ghazanfar AA. Vocal Development through Morphological Computation. PLOS Biology. 2018;16(2):e2003933. doi:10.1371/journal.pbio.2003933.

[9] Zhao L, Rad BB, Wang X. Long-Lasting Vocal Plasticity in Adult Marmoset Monkeys. Proceedings Biological Sciences. 2019;286(1905):20190817. doi:10.1098/rspb.2019.0817.

[10] Zürcher Y, Burkart JM. Evidence for Dialects in Three Captive Populations of Common Marmosets (Callithrix Jacchus). International Journal of Primatology. 2017;38(4):780–793. doi:10.1007/s10764-017-9979-4.

[11] Pomberger T, Risueno-Segovia C, Löschner J, Hage SR. Precise Motor Control Enables Rapid Flexibility in Vocal Behavior of Marmoset Monkeys. Current Biology. 2018;0(0). doi:10.1016/j.cub.2018.01.070.

[12] Takahashi DY, Narayanan DZ, Ghazanfar AA. Coupled Oscillator Dynamics of Vocal Turn-Taking in Monkeys. Current Biology. 2013;23(21):2162–2168. doi:10.1016/j.cub.2013.09.005.

[13] Takahashi DY, Fenley AR, Ghazanfar AA. Early Development of Turn-Taking with Parents Shapes Vocal Acoustics in Infant Marmoset Monkeys. Philosophical Transactions of the Royal Society B: Biological Sciences. 2016;371(1693):20150370. doi:10.1098/rstb.2015.0370.

[14] Levinson SC. Turn-Taking in Human Communication – Origins and Implications for Language Processing. Trends in Cognitive Sciences. 2016;20(1):6–14. doi:10.1016/j.tics.2015.10.010.

[15] Hasson U, Ghazanfar AA, Galantucci B, Garrod S, Keysers C. Brain-to-Brain Coupling: A Mechanism for Creating and Sharing a Social World. Trends in Cognitive Sciences. 2012;16(2):114–121. doi:10.1016/j.tics.2011.12.007.

[16] Hoffmann S, Trost L, Voigt C, Leitner S, Lemazina A, Sagunsky H, et al. Duets Recorded in the Wild Reveal That Interindividually Coordinated Motor Control Enables Cooperative Behavior. Nature Communications. 2019;10(1):2577. doi:10.1038/s41467-019-10593-3.

[17] Hasson U, Frith CD. Mirroring and beyond: Coupled Dynamics as a Generalized Framework for Modelling Social Interactions. Philosophical Transactions of the Royal Society B: Biological Sciences. 2016;doi:10.1098/rstb.2015.0366.

[18] Snowdon CT, Cleveland J. "Conversations" among Pygmy Marmosets. American Journal of Primatology. 1984;7(1):15–20. doi:10.1002/ajp.1350070104.

[19] de la Torre S, Snowdon CT. Dialects in Pygmy Marmosets? Population Variation in Call Structure. American Journal of Primatology. 2009;71(4):333–342. doi:10.1002/ajp.20657.

[20] Snowdon CT, Elowson AM. Pygmy Marmosets Modify Call Structure When Paired. Ethology. 1999;105(10):893–908. doi:10.1046/j.1439-0310.1999.00483.x.

[21] Elowson AM, Snowdon CT. Pygmy Marmosets, Cebuella Pygmaea, Modify Vocal Structure in Response to Changed Social Environment. Animal Behaviour. 1994;47(6):1267–1277. doi:10.1006/anbe.1994.1175.

[22] Hotchkin CF, Parks SE, Weiss DJ. Noise-Induced Frequency Modifications of Tamarin Vocalizations: Implications for Noise Compensation in Nonhuman Primates. PLOS ONE. 2015;10(6):e0130211. doi:10.1371/journal.pone.0130211.

[23] Candiotti A, Zuberbuhler K, Lemasson A. Convergence and Divergence in Diana Monkey Vocalizations. Biology Letters. 2012;8(3):382–385. doi:10.1098/rsbl.2011.1182.

[24] Lemasson A, Glas L, Barbu S, Lacroix A, Guilloux M, Remeuf K, et al. Youngsters Do Not Pay Attention to Conversational Rules: Is This so for Nonhuman Primates? Scientific Reports. 2011;1:22. doi:10.1038/srep00022.

[25] Lemasson A, Ouattara K, Petit EJ, Zuberbühler K. Social Learning of Vocal Structure in a Nonhuman Primate? BMC Evolutionary Biology. 2011;11:362. doi:10.1186/1471-2148-11-362.

[26] Hage SR, Gavrilov N, Nieder A. Cognitive Control of Distinct Vocalizations in Rhesus Monkeys. Journal of Cognitive Neuroscience. 2013;25(10):1692–1701.

[27] Hage SR, Gavrilov N, Nieder A. Developmental Changes of Cognitive Vocal Control in Monkeys. The Journal of Experimental Biology. 2016;219(11):1744–1749. doi:10.1242/jeb.137653.

[28] Sugiura H. Matching of Acoustic Features during the Vocal Exchange of Coo Calls by Japanese Macaques. Animal Behaviour. 1998;55(3):673–687. doi:10.1006/anbe.1997.0602.

[29] Ey E, Rahn C, Hammerschmidt K, Fischer J. Wild Female Olive Baboons Adapt Their Grunt Vocalizations to Environmental Conditions. Ethology. 2009;115(5):493–503. doi:10.1111/j.1439-0310.2009.01638.x.

[30] Levréro F, Carrete-Vega G, Herbert A, Lawabi I, Courtiol A, Willaume E, et al. Social Shaping of Voices Does Not Impair Phenotype Matching of Kinship in Mandrills. Nature Communications. 2015;6(1):7609. doi:10.1038/ncomms8609.

[31] Watson SK, Townsend SW, Schel AM, Wilke C, Wallace EK, Cheng L, et al. Vocal Learning in the Functionally Referential Food Grunts of Chimpanzees. Current Biology. 2015;25(4):495–499. doi:10.1016/j.cub.2014.12.032.

[32] Crockford C, Herbinger I, Vigilant L, Boesch C. Wild Chimpanzees Produce Group-Specific Calls: A Case for Vocal Learning? Ethology. 2004;110(3):221–243. doi:10.1111/j.1439-0310.2004.00968.x.

[33] Mitani J, Gros-Louis J. Chorusing and Call Convergence in Chimpanzees: Tests of Three Hypotheses. Behaviour. 1998;135(8):1041–1064. doi:10.1163/156853998792913483.

[34] Watts DP. Production of Grooming-Associated Sounds by Chimpanzees (Pan Troglodytes) at Ngogo: Variation, Social Learning, and Possible Functions. Primates. 2016;57(1):61–72. doi:10.1007/s10329-015-0497-8.

[35] Kalan AK, Mundry R, Boesch C. Wild Chimpanzees Modify Food Call Structure with Respect to Tree Size for a Particular Fruit Species. Animal Behaviour. 2015;101:1–9. doi:10.1016/j.anbehav.2014.12.011.

[36] Hopkins WD, Taglialatela JP, Leavens DA. Chimpanzees Differentially Produce Novel Vocalizations to Capture the Attention of a Human. Animal Behaviour. 2007;73(2):281–286. doi:10.1016/j.anbehav.2006.08.004.

[37] Bianchi S, Reyes LD, Hopkins WD, Taglialatela JP, Sherwood CC. Neocortical Grey Matter Distribution Underlying Voluntary, Flexible Vocalizations in Chimpanzees. Scientific Reports. 2016;6:34733. doi:10.1038/srep34733.

[38] van Schaik CP, Ancrenaz M, Borgen G, Galdikas B, Knott CD, Singleton I, et al. Orangutan Cultures and the Evolution of Material Culture. Science (New York, NY). 2003;299(5603):102–105. doi:10.1126/science.1078004.

[39] Wich SA, Krützen M, Lameira AR, Nater A, Arora N, Bastian ML, et al. Call Cultures in Orang-Utans? PLoS ONE. 2012;7(5):e36180. doi:10.1371/journal.pone.0036180.

[40] Wich SA, Swartz KB, Hardus ME, Lameira AR, Stromberg E, Shumaker RW. A Case of Spontaneous Acquisition of a Human Sound by an Orangutan. Primates. 2009;50(1):56–64. doi:10.1007/s10329-008-0117-y.

[41] Lameira AR, Hardus ME, Kowalsky B, de Vries H, Spruijt BM, Sterck EHM, et al. Orangutan (*Pongo* Spp.) Whistling and Implications for the Emergence of an Open-Ended Call Repertoire: A Replication and Extension. The Journal of the Acoustical Society of America. 2013;134(3):2326–2335. doi:10.1121/1.4817929.

[42] Lameira AR, Hardus ME, Bartlett AM, Shumaker RW, Wich SA, Menken SBJ. Speech-Like Rhythm in a Voiced and Voiceless Orangutan Call. PLoS ONE. 2015;10(1):e116136. doi:10.1371/journal.pone.0116136.

[43] Lameira AR, Hardus ME, Mielke A, Wich SA, Shumaker RW. Vocal Fold Control beyond the Species-Specific Repertoire in an Orang-Utan. Scientific Reports. 2016;6:30315. doi:10.1038/srep30315.

[44] Lameira AR, Shumaker RW. Orangutans Show Active Voicing through a Membranophone. Scientific Reports. 2019;9(1):1–6. doi:10.1038/s41598-019-48760-7.

[45] Hardus ME, Lameira AR, Schaik CPV, Wich SA. Tool Use in Wild Orang-Utans Modifies Sound Production: A Functionally Deceptive Innovation? Proceedings of the Royal Society of London B: Biological Sciences. 2009;276(1673):3689–3694. doi:10.1098/rspb.2009.1027.

[46] Lameira AR, Hardus ME, Wich SA. Orangutan Instrumental Gesture-Calls: Reconciling Acoustic and Gestural Speech Evolution Models. Evolutionary Biology. 2012;39(3):415–418. doi:10.1007/s11692-011-9151-6.

[47] Robbins MM, Ando C, Fawcett KA, Grueter CC, Hedwig D, Iwata Y, et al. Behavioral Variation in Gorillas: Evidence of Potential Cultural Traits. PLOS ONE. 2016;11(9):e0160483. doi:10.1371/journal.pone.0160483.

[48] Perlman M, Clark N. Learned Vocal and Breathing Behavior in an Enculturated Gorilla. Animal Cognition. 2015;18(5):1165–1179. doi:10.1007/s10071-015-0889-6.

[49] Koda H, Lemasson A, Oyakawa C, Rizaldi, Pamungkas J, Masataka N. Possible Role of Mother-Daughter Vocal Interactions on the Development of Species-Specific Song in Gibbons. PLoS ONE. 2013;8(8):e71432. doi:10.1371/journal.pone.0071432.

[50] Holy TE, Guo Z. Ultrasonic Songs of Male Mice. PLoS Biology. 2005;3(12):e386. doi:10.1371/journal.pbio.0030386.

[51] Kikusui T, Nakanishi K, Nakagawa R, Nagasawa M, Mogi K, Okanoya K. Cross Fostering Experiments Suggest That Mice Songs Are Innate. PLoS ONE. 2011;6(3):e17721. doi:10.1371/journal.pone.0017721.

[52] Arriaga G, Zhou EP, Jarvis ED. Of Mice, Birds, and Men: The Mouse Ultrasonic Song System Has Some Features Similar to Humans and Song-Learning Birds. PLoS ONE. 2012;7(10):e46610. doi:10.1371/journal.pone.0046610.

[53] Mahrt EJ, Perkel DJ, Tong L, Rubel EW, Portfors CV. Engineered Deafness Reveals That Mouse Courtship Vocalizations Do Not Require Auditory Experience. Journal of Neuroscience. 2013;33(13):5573–5583. doi:10.1523/JNEUROSCI.5054-12.2013.

[54] Arriaga G, Jarvis ED. Mouse Vocal Communication System: Are Ultrasounds Learned or Innate? Brain and language. 2013;124(1). doi:10.1016/j.bandl.2012.10.002.

[55] Grimsley JMS, Monaghan JJM, Wenstrup JJ. Development of Social Vocalizations in Mice. PLoS ONE. 2011;6(3):e17460. doi:10.1371/journal.pone.0017460.

[56] Miller JR, Engstrom MD. Vocal Stereotypy and Singing Behavior in Baiomyine Mice. Journal of Mammalogy. 2007;88(6):1447–1465. doi:10.1644/06-MAMM-A-386R.1.

[57] Okobi DE, Banerjee A, Matheson AMM, Phelps SM, Long MA. Motor Cortical Control of Vocal Interaction in Neotropical Singing Mice. Science. 2019;363(6430):983–988. doi:10.1126/science.aau9480.

[58] Long CV. Vocalisations of the Defu *OCTODON DEGUS* ,a Social Caviomorph Rodent. Bioacoustics. 2007;16(3):223–244. doi:10.1080/09524622.2007.9753579.

[59] Tokimoto N, Okanoya K. Spontaneous Construction of "Chinese Boxes" by Degus (*Octodon Degu*): A Rudiment of Recursive Intelligence?: Cup Nesting by Degus. Japanese Psychological Research. 2004;46(3):255–261. doi:10.1111/j.1468-5584.2004.00257.x.

[60] Lidhar NK, Insel N, Dong JY, Takehara-Nishiuchi K. Observational Fear Learning in Degus Is Correlated with Temporal Vocalization Patterns. Behavioural Brain Research. 2017;332:362–371. doi:10.1016/j.bbr.2017.06.011.

[61] Vanden Hole C, Van Daele PAAG, Desmet N, Devos P, Adriaens D. Does Sociality Imply a Complex Vocal Communication System? A Case Study for *Fukomys Micklemi* (Bathyergidae, Rodentia). Bioacoustics. 2014;23(2):143–160. doi:10.1080/09524622.2013.841085.
